# MicroRNAs Regulating Signaling Pathways: Potential Biomarkers in Systemic Sclerosis

**DOI:** 10.1016/j.gpb.2015.07.001

**Published:** 2015-09-11

**Authors:** Yisha Li, Jing Huang, Muyao Guo, Xiaoxia Zuo

**Affiliations:** Department of Rheumatology, Xiangya Hospital, Central South University, Changsha 410008, China

**Keywords:** MicroRNA, Systemic sclerosis, Signaling pathway, TGF-β, Microvascular endothelial cell, Toll-like receptor

## Abstract

**Systemic sclerosis** (SSc) is a multisystem fibrotic and autoimmune disease. Both genetic and epigenetic elements mediate SSc pathophysiology. This review summarizes the role of one epigenetic element, known as **microRNAs** (miRNAs), involved in different **signaling pathways** of SSc pathogenesis. The expression of key components in transforming growth factor-β (**TGF-β**) **signaling pathway** has been found to be regulated by miRNAs both upstream and downstream of **TGF-β**. We are specifically interested in the pathway components upstream of **TGF-β**, while miRNAs in other **signaling pathways** have not been extensively studied. The emerging role of miRNAs in vasculopathy of SSc suggests a promising new direction for future investigation. Elucidation of the regulatory role of miRNAs in the expression of signaling factors may facilitate the discovery of novel biomarkers in SSc and improve the understanding and treatment of this disease.

## Introduction

Systemic sclerosis (SSc) is a multisystem fibrotic and autoimmune disease mediated by complex interactions between endothelial damage, non-infective inflammation or autoimmunity, and fibroblast to myofibroblast transition [1,2]. Endothelial damage may be the primary event in the pathogenesis of SSc, resulting in narrowing of the capillary lumen and loss of the microvasculature, while fibrosis appears to be the final consequence [2–6].

The immune system participates in the pathogenesis of SSc. Adaptive immune cells, particularly T cells, which were found to be adjacent to myofibroblasts in affected tissues of SSc patients, are self-intolerant, and secrete pro-inflammatory and fibrogenic cytokines [1]. Furthermore, T helper (Th) cell balance is biased toward Th2 in SSc patients. There is a higher level of Th2 cytokines in the dermal fibroblasts, such as interleukin (IL)-13, which could induce collagen expression [1–3,5,6]. B cells are not generally prominent in SSc lesions but are generally activated, resulting in the autoimmune response [2,3]. Monocytes of the innate immune system appear to initially infiltrate the SSc lesions, differentiate into dendritic cells upon lesional stimulation, and generate profibrotic cytokines [1].

Excessive fibrogenesis is a prominent occurrence in SSc pathogenesis. Soluble mediators generated by the endothelial and inflammatory cells suggest inappropriate activation of fibroblasts, resulting in the secretion of extracellular matrix (ECM) macromolecules, growth factors, and cytokines, as well as transdifferentiation into contractile myofibroblasts [2–8]. Myofibroblasts are effector cells in SSc fibrosis, which either produce excessive fibrillar collagens in the accumulated ECMs or disrupt the normal tissue architecture, leading to the loss of organ function [1,2,9].

Compared to genetic factors, epigenetics plays a much larger role in SSc pathogenesis [10–14]. Epigenetic processes are adaptive mechanisms responding to environmental stimuli. These include DNA methylation (of cytosine at position C5 at CpG dinucleotides), histone modifications (lysine acetylation, lysine and arginine methylation, serine and threonine phosphorylation, lysine ubiquitination and sumoylation), and RNA interference (including gene silencing induced by microRNAs, long non-coding RNAs, RNA modifications, *etc*.). Studies suggest that these aforementioned epigenetic processes do occur in SSc pathogenesis [10–13,15].

MicroRNAs (miRNAs) are small non-coding RNAs (ncRNAs) approximately 21 nucleotides in length that are partially or completely complementary to and bind with mRNAs, leading to mRNA degradation or reduced mRNA translation. miRNAs account for 1%–2% of the human genome [10]; they are transcribed as precursors and further cleaved into mature miRNAs by ribonuclease Drosha/DGCR-8, and Dicer in the nucleus and cytoplasm [10]. Differentially-expressed miRNAs identified in SSc target both inflammation and fibrosis [10,14]. Functional studies suggest that miRNAs mainly modulate fibrosis-related genes encoding collagens, metallopeptidases, and integrins [11,16–20].

## Differentially-expressed miRNA profile in SSc

### Downregulated miRNAs

Downregulated miRNAs primarily serve as inhibitory miRNAs for fibrotic genes. MiR-29a and miR-29b have been identified as targets for downregulation in dermal fibroblasts from SSc patients and bleomycin-induced SSc mice [21,22] and, interestingly, in hair samples from SSc patients [23]. However, such downregulation was not observed in the serum of SSc patients [24]. Numerous studies have supported the role of miR-29s in SSc pulmonary fibrosis [25,26] and in phalangeal contracture [23]. miR-196a is also reportedly downregulated in dermal fibroblasts and serum of SSc patients [27,28]. Moreover, miR-196a levels in hair shafts are stably decreased in SSc patients, indicating that this miRNA can be used as a new biomarker for SSc diagnosis [29]. Similarly, miR-150 was downregulated in dermal fibroblasts and serum of SSc patients as well, and its level was negatively correlated with disease severity [30]. Let-7a is another miRNA that is downregulated miRNA in both dermal fibroblasts and serum of SSc patients [31]. Additional miRNAs downregulated in SSc dermal fibroblasts or microvascular endothelial cells (MVECs) include miR-129-5p, miR-30b, miR-145, miR-193b, and miR-152 [22,32,33]. Among them, levels of miR-30b were inversely correlated with modified Rodnan skin scores of these patients, reflecting disease severity [33]. Other than those, downregulation of miR-223 has been demonstrated in SSc plasma samples [34].

### Upregulated miRNAs

The upregulated miRNAs mostly inhibit anti-fibrotic genes. Our group found that miR-21 was upregulated in dermal fibroblasts and skin tissues of SSc patients based on microarray analysis; miR-130b, miR-146, and miR-503, among others, were also upregulated in skin tissues [22]. Furthermore, the elevated expression of miR-146 in skin samples may be correlated with the occurrence of vascular abnormality in SSc, such as telangiectasia [35]. The elevated miR-92a levels in serum and in dermal fibroblasts of SSc patients were also correlated with the presence of telangiectasia but not with disease activity [36].

### miRNAs with conflicting findings

There are some miRNAs with conflicting findings in their expression levels in SSc. Their roles in SSc pathogenesis need to be further investigated. For example, miR-7 targets fibrotic genes, but is upregulated in SSc. One microarray analysis indicated that the miR-7 level was upregulated in SSc fibroblasts, suppressing fibrotic processes. This was suggested to be a negative feedback loop against excessive fibrogenesis [37] but fail to overcome the pathogenic fibrosis in SSc. Another study reported that miR-7 level was downregulated in localized scleroderma dermis but not in SSc dermis, suggesting that miR-7 may also target unknown anti-fibrotic pathways and act as a modulator in both sides of fibrosis regulation [38].

Similarly, conflicting findings were also documented for miR-142-3p. One report demonstrated significant upregulation of miR-142-3p in the serum of SSc patients, particularly in those with a short lingual frenulum [39]. This finding suggests a possible negative feedback effect of miR-142-3p on over-activation of tumor growth factor-β (TGF-β) in SSc dermal fibroblasts. However, another report suggested that level of this anti-fibrotic miRNA was downregulated in the serum of SSc patients, in comparison to systemic lupus erythematosus patients and healthy controls [34]. These findings suggest that miR-142-3p may be another miRNA with dual effects in fibrosis regulation.

### miRNAs in TGF-β signaling pathway in SSc

#### TGF-β signaling in SSc

The TGF-β pathway is a critical signaling pathway in fibrosis, particularly SSc. TGF-β is secreted as a latent precursor and requires proteolytic cleavage for activation by integrins, thrombospondin-1, or reactive oxygen species [1,4,6,8]. For TGF-β pathway, the downstream activation of the canonical SMAD pathway (SMAD2/3), STAT3, and other noncanonical pathways (*e.g.*, SMAD1, c-Abl, EGR1, and ERK1/2) is associated with the profibrotic response and induction of myofibroblast differentiation [1,2,4,6–9]. In SSc fibroblasts, multiple abnormalities occur in the SMAD signaling pathways. These include elevated expression or phosphorylation of SMAD2/3, constitutive association of SMAD2/3 with its coactivator p300, elevated levels of p300, and defective function of SMAD7, the endogenous inhibitor of SMAD signaling [8,40,41]. Activation of non-canonical signaling pathways may occur via autocrine TGF-β stimulation, including SMAD1, AKT, and ERK1/ERK2, contributing to the maintenance of the activated fibroblast phenotype even in the absence of exogenous stimuli [4,8]. TGF-β further upregulates the expression of connective-tissue growth factor (CTGF) [6], which possesses profibrotic activities similar to TGF-β, and downregulates the levels of IL-17A receptor, thus inhibiting the suppressive impact of IL-17A on CTGF and collagen expression [32]. Interestingly, myofibroblast contraction can mechanically activate the TGF-β precursor, reinforcing this loop [1,3,9].

### miRNAs targeting factors downstream of TGF-β

The miR-29 family members, including miR-29a and miR-29b, are the most important miRNAs involved in TGF-β signaling and collagen production [21,42]. Recently, TGF-β activated kinase 1 (TAK1)-binding protein 1 (TAB1) was defined as a new miR-29a target. Upregulation of TAB1 expression was found to trigger the production of the tissue inhibitor of metalloproteinase-1 (TIMP-1) and inhibition of matrix metalloproteinase (MMP)-1, leading to reduced collagen degradation [43]. TGF-β, platelet-derived growth factor (PDGF), and IL-4 have been identified as upstream modulators of miR-29 members in SSc, which inhibit transcription of miR-29 members [21,42,44]. miR-29a expression is downregulated by SMAD3 via TGF-β/SMAD signaling in SSc, which has been confirmed by protection against bleomycin or TGF-β1-induced loss of miR-29a along with fibrosis in SMAD3-null models [25].

A group of miRNAs in SSc pathogenesis directly targets collagen genes and is inhibited by upstream TGF-β signaling. For example, miR-196a is negatively modulated by TGF-β and targets transcripts encoding collagen in fibroblasts, particularly collagen 1 and collagen 3 [27,28,45]. Additionally, let-7a affected the expression of type I collagen as an upstream modulator and in turn was downregulated by active TGF-β signaling [31]. miR-129-5p expression is downregulated by TGF-β signaling by suppressing IL-17A signaling, thus leading to increased α1(I) collagen expression [32].

Another group of miRNAs targets the downstream factors of the TGF-β signaling pathway, such as SMADs. Our group found that the upregulated miR-21 in SSc targeted SMAD7, which was verified using a reporter gene assay [22,46]. As described above, SMAD7 is an endogenous inhibitor of SMAD signaling. Therefore, downregulation of SMAD7 by miR-21 leads to increased SMAD signaling followed by fibrosis. miR-21 is thought to be upregulated by TGF-β in SSc pathogenesis [22]. There are other miRNAs that target SMADs downstream of TGF-β. The putative targets of miR-145, miR-146, and miR-503 are SMAD3, SMAD4, and SMAD7, respectively, which requires further verification [22].

### miRNA targeting factors upstream of TGF-β

The major upstream factors of TGF-β targeted by miRNAs include integrins, which are activators of TGF-β precursors. miR-150 targets integrin-β3, a key upstream event in TGF-β activation, followed by phosphorylation of SMAD3 and accumulation of type I collagen [30]. Downregulation of miR-150 in SSc may occur through an epigenetic mechanism and involves DNA methylation [30]. Protein expression of laminin and integrin was also significantly elevated in miR-29-knockdown fibroblasts, similar to collagen genes [26]. Therefore, miR-29 may target integrin genes, suggesting that it is also an upstream modulator of TGF-β activation. miR-142-3p is one of the miRNAs regulating the expression of integrin αV [39]. Decreased expression of miR-142-3p leads to overexpression of integrin αV and consequently TGF-β over-activation in SSc dermal fibroblasts [47,48].

### miRNAs in vasculopathy signaling pathways in SSc

#### The uPA signaling pathway

Urokinase-type plasminogen activator (uPA) and its receptor (uPAR) pathway in vasculopathy facilitate neointimal growth and vascular remodeling, as well as fibrosis by enhancing matrix degradation [49,50]. Recent studies have confirmed that impaired uPA/uPAR signaling caused by inactivation and cleavage of uPARs in MVECs contributed to poor angiogenesis in SSc pathogenesis [51,52]. However, uPA levels were shown to be elevated in SSc dermal fibroblasts in response to TGF-β stimulation [51,52]. The enhanced uPA signaling induced cell proliferation and inhibited apoptosis of human pulmonary artery smooth muscle cells via uPAR-sparing pathways, leading to proliferative vasculopathy in SSc [51,52]. Furthermore, Iwamoto et al. [53] find out that miR-193b targets uPA and is downregulated in SSc dermal fibroblasts, possibly accounting for the upregulation of uPA expression. Thus, miR-193b downregulation probably contributes to proliferative vasculopathy in SSc.

#### DNA methyltransferase 1

Evidence suggests that miR-152 targeting of DNA methyltransferase 1 (DNMT1) leads to decreased global DNA methylation, including the genes encoding the bone morphogenic protein (BMP) receptor II and nitric oxide synthetase (NOS) [54]. Downregulation of miR-152 and the resulting gene hypermethylation in SSc MVECs lead to endothelial cell (EC) apoptosis, vasoconstriction, inflammatory cell infiltration, and ultimately fibroblast activation [54]. The relationship between miR-152 and the signal molecules secreted by damaged ECs in SSc requires further analysis.

### miRNAs in TLR signaling in SSc

#### TLR signaling in SSc

The role of innate immune signaling and Toll-like receptors (TLRs), particularly TLR4, signaling in SSc fibrosis have been examined in recent decades [55]. Endogenous ligands for TLRs in SSc include ECM molecules (*e.g.*, alternatively-spliced fibronectins, abbreviated as Fn-EDA, induced by TGF-β in normal fibroblasts), cellular stress proteins (high-mobility group protein B1, abbreviated as HMGB1, and HSP60), and nucleic-acid-containing immune complexes [4,7,55]. TLR signaling, particularly TLR4, results in inflammatory cell infiltration, B cell activation, polarization toward a Th2/Th17 response, activation of nuclear factor kappa beta (NF-κB), elevated expression of profibrotic cytokines (*e.g.*, IL-4 and IL-6), activation of TGF-β downstream canonical SMAD signaling, and pathological angiogenesis in SSc [55–60]. The expression of TLR4, its ligands (*e.g.*, S100A8/A9), and its co-receptors (MD2 and CD14) was elevated in the plasma, lesional skin, and lung biopsies of SSc patients [55,58,61]. TLR8 on SSc monocytes recognizes immune complexes against self-DNA/RNA to trigger activation of NF-κB and downstream genes, such as interferon [1,55]. This leads to the production of profibrotic TIMP-1 and enhances ECM deposition via an MMP blockade in an IL-1R-associated kinase (IRAK)-dependent mechanism [1,55,57,62]. Additional evidence suggests that TLR2, TLR3, TLR5, TLR7, TLR9, and TLR10 mediate SSc pathogenesis, although their involvement remains controversial [4,55,57,63–66].

### miRNAs and TLR signaling pathway

The evidence is very limited for direct regulation of miRNAs in TLR signalings. miR-29 is the only one definitely reported in the literature so far. Activated TLR4 signaling targets antifibrotic miR-29, leading to its downregulation along with SMAD2/3 upregulation [55,58]. Both of these downstream signals contribute to fibrotic processes in SSc. TLR signaling pathway is very important in SSc pathogenesis and displays close interaction with TGF-β downstream signaling. As mentioned above, a large number of miRNAs participated in TGF-β downstream signaling pathways. These miRNAs target the common downstream components in both pathways and thus may also influence TLR downstream signaling as well.

### Epigenetic regulation of Wnt signaling in SSc

The Wnt signaling pathway is involved in SSc pathogenesis, particularly fibrosis, in addition to TGF-β signaling. Binding of Wnt to the surface receptor frizzled stabilizes and activates β-catenin in the canonical TGF-β pathway. The activated β-catenin translocates to the nucleus and activates target genes in TGF-β signaling [1,7,8]. In addition to the induction of TGF-β signaling, nuclear β-catenin also activates the myofibroblast transition [1,3,4,8,67,68]. In skin biopsies of SSc patients and scleroderma mice, evidence supports constitutively-activated Wnt-β-catenin signaling (Wnt1, Wnt3a, and Wnt10b) and elevated expression of Wnt2, Wnt9a, Wnt11, and secreted frizzled-related proteins (SFRP) [8,67–70]. Epigenetic suppression of Wnt antagonists occurs in the fibroblasts and peripheral blood mononuclear cells of SSc patients [71,72]. However, there is still no supporting evidence for the involvement of miRNAs in this signaling pathway in SSc. Therefore, further investigation is required.

### miRNAs in other signaling pathways in SSc

The precise target of miR-92a has not been identified. However, evidence suggests that miR-92a downregulates the protein expression of MMP-1, an enzyme that degrades the ECM [36]. TGF-β may be an upstream modulator of miR-92a [36]. The levels of miR-92a are significantly elevated in SSc, leading to MMP-1 downregulation followed by reduced ECM degradation. In our previous study, the target of miR-130b was found to be peroxisome proliferator-activated receptors (PPAR), which disrupt TGF-β signaling through an anti-fibrotic signaling mechanism [73,74]. Upregulation of miR-130b expression in SSc may result in defective PPAR expression and ultimately enhance TGF-β signaling and fibrosis-related gene expression. Expression of miR-130b is upregulated by TGF-β via the TGF-β/SMAD pathway, suggesting a positive feedback route for over-activation of TGF-β signaling [73].

Another signaling pathway, platelet-derived growth factor (PDGF) pathway, is related to SSc pathogenesis and is the target for some miRNAs. Transfection of miR-30b repressed the expression of PDGF receptor (PDGFR)-β in dermal fibroblasts by directly binding to the *PDGFRB* mRNA, which was verified using luciferase reporter gene assays [33]. Therefore, miR-30b likely mediates SSc pathogenesis via the PDGF signaling pathway.

As described above, miR-196a targets genes coding for collagens. The possible feedback to excessive collagen accumulation is mediated via the discoidin domain receptor 2 (DDR2)-miR-196a pathway [75]. In SSc fibroblasts, this pathway may be impaired, resulting in the abnormal accumulation of ECMs [75].

For miR-7, it is an upstream downregulator of type I collagen mRNAs [37,38]. miR-7 level was upregulated in SSc fibroblasts, showing a negative feedback effect against fibrosis due to its downregulated suppressor, intracellular TSP-2 [37,38]. However, expression of miR-7 was downregulated in localized scleroderma dermis, suggesting modulation via unknown pathways, leading to overexpression of collagens in SSc pathogenesis [38].

## Discussion

SSc phenotypes display marked heterogeneity and a complex interplay between different pathogenic processes, including vasculopathy, autoimmunity, and fibrosis. The key challenge in clinical practice is the discovery of new biomarkers that can be used for better diagnosis and disease classification. Notably, miRNAs are involved in different signaling pathways of SSc and regulate its pathophysiology, suggesting their promising role as biomarkers of SSc.

**Figure 1:**
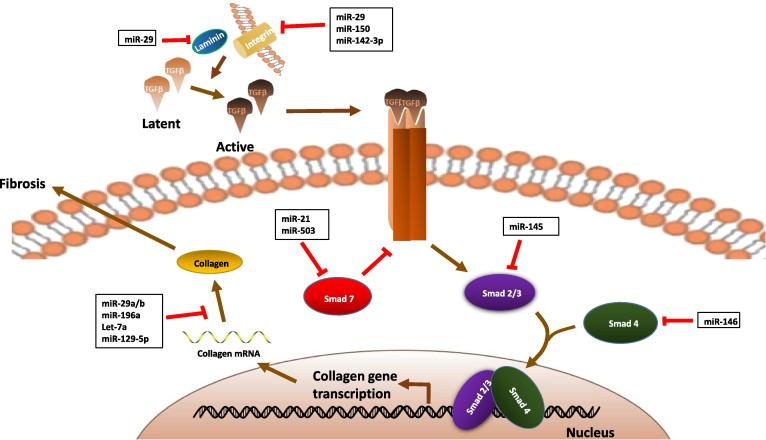
**miRNA participation in TGF-β signaling pathway in systemic sclerosis**

Among the miRNAs that mediate SSc pathogenesis via different signaling pathways (Table 1), TGF-β signaling is the most important in SSc fibrosis and the involvement of miRNAs in this pathway has been extensively studied (Figure 1). The mechanisms of other signaling pathways and miRNAs remain unclear. As evidence supporting miRNA functioning in vasculopathy of SSc accumulates, we hypothesize that this primary event in SSc pathogenesis is controlled by a complex miRNA network. Therefore, several unresolved issues regarding miRNA regulation and the roles of miRNAs in different signaling pathways should be examined to gain a better understanding of SSc pathogenesis.

miRNAs have recently emerged as promising biomarkers of various autoimmune disorders, such as systemic lupus erythematosus, rheumatoid arthritis, type 1 diabetes mellitus, and multiple sclerosis [76]. The roles of miRNAs overlap in different autoimmune disorders, suggesting common pathways in their pathogenesis. Therefore, some miRNAs mediating SSc pathogenesis are summarized in this review, including miR-21 and miR-146, among others [76]. Expression of miR-21 is upregulated in SSc patients similarly as in SLE patients’ CD4^+^ T cells [77]. However, miRNAs, which may share similar pathways, are differentially regulated based on the clinical heterogeneity of individual disorders. For instance, SLE patients had reduced miR-146a levels [78], whereas SSc patients showed an upregulated expression of miR-146a [22], possibly because of the Th2 bias phenomenon in SSc pathogenesis. miR-146a in PBMCs of the Chinese lupus population suppresses the interferon pathway [79] and the Th1 response [80].

In this review, we summarized miRNAs that have been reported to be involved in SSc pathogenic pathways to highlight their role in the pathogenesis of this disease and to understand the interaction of different signaling pathways in the same disease. For SSc, different phenotypes have characteristic features in signaling activation, which could be partially attributed to miRNA interference. Therefore, miRNAs can be potential biomarkers for early discovery of onsets of severe phenotypes. The miRNA signatures are both uniform (in terms of similar signaling pathways involved) and heterogeneous (in terms of individual performance of these pathways in different diseases) for different autoimmune diseases, therefore they are promising biomarkers for differential diagnosis of these diseases. However, many signaling pathways in SSc pathogenesis have not been clearly described in terms of miRNA interference. Are there new miRNA players involved in the regulation of these pathways? Are these miRNAs associated with different SSc phenotypes? Further efforts are needed to answer these questions.

## Competing interests

The authors have declared no competing interests.

## Figures and Tables

**Table 1:** miRNA expression in systemic sclerosis

**miRNA**	**Expression**	**Target genes**	**Refs.**
*TGF-β downstream signaling*			
miR-29a	Downregulated	*COL1A1, COL3A1*	[21]
miR-29b	Downregulated	*COL1A1*	[42]
miR-196a	Downregulated	*COL1A1, COL3A1*	[45]
let-7a	Downregulated	*COL1A1, COL1A2*	[31]
miR-129-5p	Downregulated	*COL1A1*	[32]
miR-21	Upregulated	*SMAD7*	[22,46]
miR-145	Downregulated	*SMAD3*	[22]
miR-146	Upregulated	*SMAD4*	[22]
miR-503	Upregulated	*SMAD7*	[22]

*TGF-β upstream regulator*			
miR-150	Downregulated	*ITGB3*	[30]
miR-142-3p	Controversial	*ITGAV*	[39]
miR-29	Downregulated	Laminin genes and integrin genes	[26]

*uPA signaling in MVECs*			
miR-193b	Downregulated	*PLAU*	[53]

*Hypermethylation in MVECs*			
miR-152	Downregulated	*DNMT1*	[54]

*TLR signaling*			
miR-29	Downregulated	*COL1A1, COL3A1*	[21]

*PDGF signaling*			
miR-30b	Downregulated	*PDGFRB*	[33]

*TSP-2 signaling*			
miR-7	Controversial	*COL1A2*	[38]

*Other signaling*			
miR-92a	Upregulated	*MMP1*	[36]
miR-130b	Upregulated	*PPARG*	[73,74]
